# MPLA case: How do you lead as a lead physicist?

**DOI:** 10.1002/acm2.13994

**Published:** 2023-04-13

**Authors:** Patricia Sansourekidou, Leonard Kim, Lee Xu, Mary Gronberg, Cassandra Stambaugh, Dongxu Wang

**Affiliations:** ^1^ Department of Radiation Oncology University of New Mexico Albuquerque New Mexico USA; ^2^ MD Anderson Cancer Center at Cooper and Cooper Medical School of Rowan University Camden New Jersey USA; ^3^ New York Proton Center New York New York USA; ^4^ University of Texas MD Anderson Cancer Center Houston Texas USA; ^5^ Department of Radiation Oncology Tufts Medical Center Boston Massachusetts USA; ^6^ Department of Medical Physics Memorial Sloan Kettering Cancer Center New York New York USA

**Keywords:** case study, leadership, MPLA, professionalism

## Abstract

This work of fiction is part of a case study series developed by the Medical Physics Leadership Academy (MPLA). It is intended to facilitate the discussion of the managerial and leadership challenges faced by a clinical medical physicist. In this case, a physicist David used to work in a clinic where he thrived and felt like a leader, despite not having the title. After a job change, he is now officially the “Lead Physicist” at a hospital newly affiliated with a large academic healthcare system. He believes he will be equally successful. Yet he struggles to bring about changes and get buy‐in from coworkers. In the end, he feels like giving up and considers changing his job. This case is in the scenario of Problem Diagnosis.^i^ The intended use of this case, through group discussion or self‐study, is to encourage readers to perform a comprehensive analysis that identifies the root cause of the problem. This case study falls under the scope of and is supported by the MPLA, a committee in the American Association of Physicists in Medicine (AAPM).

## 1 INTRODUCTION AND CASE NARRATIVE

*This work of fiction is part of a case study series developed by the Medical Physics Leadership Academy (MPLA). The intended use of this case, through group discussion or self‐study, is to encourage readers to discuss the situation at hand and inspire professionalism and leadership thinking. This case study falls under the scope of and is supported by the* ^i^ MPLA*, a committee in the American Association of Physicists in Medicine (AAPM)*.

David was a medical physicist at a small community hospital in New Troy. He loved his job and the people he worked with. Everyday he went to work feeling happy that he was making a difference. The department had a culture where a physicist's opinion was taken seriously—David frequently took charge of things and felt well‐respected doing so. Despite not having an official leadership title, David was seen as the “change agent” in his department and wasn't afraid to speak up. However, one aspect he didn't like about his job was that the machines in the department were very old, and administration was reluctant to invest in upgrades. David constantly felt like he was falling behind the technology curve. One afternoon, he started looking for jobs at other hospitals just to see what was out there. He applied to a few, and as luck would have it, got a call from the University of Liberty State Cancer Center (ULSCC), which was part of the largest health system in the state. David was being considered for the Lead Physicist position at Red Sands Hospital, a newly affiliated partner hospital of ULSCC.

On the day of his interview, David met with almost everyone at Red Sands as well as the leadership from ULSCC. ULSCC was looking for someone with the leadership skills to bring Red Sands into the 21st century. The radiation oncologists at Red Sands seemed eager for change, and the department would soon be getting two new accelerators and a complete equipment refresh. By the end of the day, David was enthusiastic about the opportunity and believed that joining ULSCC would be a good step in his career. Plus, now he would finally be recognized with an official leadership title for the kind of work he'd already been doing for years at New Troy. David did sense some apathy from the Red Sands chief therapist Mark, but he figured he would easily develop a similar kind of working relationship with the chief therapist as he had at New Troy. David realized that he would be working with different personalities at Red Sands than those at New Troy, but he was certain he could handle it. The ULSCC administrators told him they believed in his ability to handle the “difficult people” at Red Sands and bring them in line with ULSCC. David accepted his appointment from ULSCC and took the job as Lead Physicist at Red Sands. He felt fortunate for the opportunity and was ready to lead the change.

On the first day of his new job, David had the usual and expected login problems. He asked one of the other physicists how to call IT. “I don't know,” the physicist said. “I usually just tell Mark.” Mark—the chief therapist—had apparently been the go‐to person for everything under the sun for the past 20 years. David was perplexed as to why a fellow physicist didn't just call IT on their own instead of having to ask the chief therapist for help. This was very strange to him, but he let it slide. Later that day, David figured out how to open IT tickets on his own and decided to e‐mail the other physicists telling them to do likewise in the future for better documentation and organization.

**LEARNING OBJECTIVES (LO)^ii^**

1. Develop organizational awareness and recognize one's appointed and implied roles in an organization.
2. Identify the importance of service orientation in teamwork and collaboration with other healthcare professionals.
3. Understand how a medical physicist may exercise influence and implement changes in a clinical setting.
4. Assess key communication skills and interpersonal skills to succeed as a physicist leader.

A few days later, David decided his big opportunity to make a mark at Red Sands was in the upcoming inspection for national re‐accreditation. He had worked on national accreditation many times before. At New Troy, David was known as “the policy king,” and he felt that getting his ideas, and more importantly the ULSCC standards, into Red Sands policies and procedures would result in both a smooth transition and successful inspection. He pulled out his old checklists, obtained the ULSCC documents, and went to work. Within a couple of weeks, David identified all the accreditation “weak spots” within the department as well as areas where Red Sands differed from ULSCC. He made a list of the issues and took it to Dr. Halpern, the medical director at Red Sands, to discuss.

“Oh don't worry about the inspection,” Dr. Halpern said. “Mark always makes us pass.”

“That may have been fine in the past, but my name is going to be on the paperwork submitted to the inspectors, and we are part of ULSCC now, so I really need to be involved at every step,” David explained.

Dr. Halpern appeared reluctant but conceded: “Let me ask Mark to forward what he has to you, and we can discuss it later. How about that?”

A month later, David still had not received the documents he was supposed to be reviewing and revising. He tried not to be pushy, but every time he mentioned it to Mark, Mark would say something like, “Oh, sorry they are on a thumb drive,” or “I have some on my laptop,” or “They are saved on the sim computer; I just need to find them.” Eventually, David got a hold of some department paper copies on his own, scanned them, converted the images to text, and, after a couple of hours of reformatting, finally had all the policies neatly organized. Over the next few weeks, he wrote and wrote, collating the documents into cohesive standard operating procedures (SOPs). Once completed, he sent the document to his supervisor at ULSCC, who promptly replied with support and approval. David then sent out an email to everyone in his Red Sands department, detailing the changes and welcoming comments. Over the next couple of weeks, he received feedback from a few of the doctors, physicists, dosimetrists, and therapists, but nothing from Mark. David found this lack of response unusual. Ideally, Mark and David would have the same great working relationship David had cultivated with his previous chief therapist, but Mark seemed wholly apathetic to anything David suggested.

Still, David didn't think the paperwork for national re‐accreditation should be submitted until the policies and procedures were agreed upon by all key members of the department, including Mark. In particular, David wanted Mark's blessing on a new immobilization procedure he'd written. David had noticed Mark did CT simulations in the same way he did 20 years ago: electron setups without any immobilization at all, pelvis scans with knee wedges instead of Vac‐Loks, etc. This may have been fine in a previous era, but patients being treated with today's complex techniques needed to be immobilized more accurately and reproducibly. The department had the equipment; they were just not being used. David asked the other physicists their opinion. They expressed agreement, but also said they didn't know if it should be their job to change anything either in the simulation room or on the treatment floor.

David tried making suggestions to Mark when they happened to be together at CT sims, but felt he was being ignored. He tried bringing it up to the radiation oncologists, who despite agreeing with him, also advocated for Mark. “Mark is a great therapist. You can trust his judgment for balancing technical precision and patient experience,” they said. David didn't necessarily disagree; Mark was a very good therapist who went out of his way for the patients. But Mark had not had any training or instruction in modern simulation and setup methods. David decided that perhaps education would be a good approach to getting Mark on‐board and e‐mailed some papers to him about inter‐fractional motion, residual error, and the importance of proper immobilization. The next time he saw Mark, he asked, “Did you get the papers I sent you?”

“Yeah.”

“Did you get a chance to read them?”

“Not yet. I've been busy treating patients.”

“I think you'll find them really interesting, and if you have any questions, I am always happy to discuss them with you.” From the lack of response to his previous emails, David gathered Mark had been busy, but David still hoped to demonstrate that research and continued training were important for ensuring the best treatment.

“OK, thanks,” Mark replied.

A month later, having heard nothing more from Mark, and with the inspection for national re‐accreditation imminent, David removed his updated simulation section from the SOP documents he had been writing, and put back Mark's original version.

Fortunately, the site passed inspection with flying colors with everyone putting on a good show. The department's accreditation was renewed, and congratulations and acknowledgements went all around. But David knew that after the inspectors left, he still had to make sure the modified policies and procedures went into action. One morning, as he was walking past the Linac console, he overheard two therapists debating what images to take for a complicated case. David stopped and said, “Oh we have an updated SOP for that. Didn't Mark tell you?”

The therapists paused their conversation. “What?” one of them said.

“We updated a section of the imaging procedure that exactly covers this topic for inspection,” David said.

“Oh, those were just for inspection. We weren't told to do anything differently.”

As soon as he could, David went straight to Mark's office. “You didn't tell your staff to use the new imaging procedure?”

“They're just for inspection. They aren't official hospital policies.”

“What do you mean?” said David. “They are our department's policies and procedures, and they're what ULSCC expects us to do.” Despite maintaining a cool exterior, David was perplexed as to why Mark was insistent that the documents he had spent weeks aligning and rewriting for approval were not important enough to implement to his team.

“Admin said we don't have to follow ULSCC policies if it's what's best for our patients and department.”

“Dr. Halpern said he wants us to follow them,” David insisted.

“Oh yeah? Do you remember that e‐mail you sent to everyone about how MDs were going to start digitally signing plans? Is Dr. Halpern following that?”

“Well, he said he wanted me to walk him through it first.”

“And you sent out that e‐mail when? 2 months ago?”

“He's a busy guy.”

“I've worked with Jim for 20 years. He'll always say he supports ‘quality improvement’,” Mark said, making finger quotes. “But neither he nor anyone else here is going to make changes we're not comfortable with. You seem to think I'm the only reason the department doesn't treat your every e‐mail like law. I'm not the reason.” Mark paused briefly before continuing. “I have to go treat our patients now.” With that, Mark excused himself, leaving David alone in the chief therapist's office.

When he got back to his own office, David wrote a long email to his ULSCC supervisor. The email was worded carefully; David wanted to balance his frustration with objectivity. Several days later, he got a brief reply. His supervisor assured him he had her and ULSCC's support, and she would like to schedule a meeting soon to discuss how to develop his leadership skills and implement changes. The supervisor's email ended with a link to some AAPM materials that he “might find useful” on how to become an influential leader.^1^, ^2^, ^3^ Her response was appreciated, but David wasn't sure he had the energy needed to bring change to Red Sands or to himself, if that's what his supervisor was implying. He thought about clicking the AAPM link his supervisor just sent him, when his e‐mail alert pinged again. It was the AAPM Career Services Update. David paused on what he was about to click, and instead clicked on “Jobs.”

## 2 DISCUSSION

The following are suggested discussion questions in relation to the learning objectives (LOs):

1. What is David's self‐identified role in his new position? What do you think are the expected roles for him from the perspectives of ULSCC and Red Sands, respectively? Are they all consistent with one another? [LO1, LO3].
2. From Question 1 above, if a person has competing roles within an organization, what could be done? [LO1, LO2].
3. David's approach to preparing for an inspection was based on his previous successes at New Troy and expectations from ULSCC. He tried to change the practices at Red Sands through the preparation for the inspection. Why was he ineffective in bringing about changes? [LO2, LO4].
4. At one point, David tried to influence the chief therapist to adopt new immobilization devices and simulation procedures. How could he have been more successful in this effort? [LO3, LO4].
5. If David were to start over again, what should he do differently? [LO1, LO2, LO3, LO4].

Sample answers to the suggested discussion questions are available in the Supplemental Materials, or by writing to the MPLA Cases Subcommittee: https://www.aapm.org/org/structure/default.asp?committee_code=MPLACA.

## 3 CONCLUSION

Case study participants should leave the discussion with an appreciation for the LOs chosen for this case study session and consider the real‐life applications in their own workplace setting or career situation.

## AUTHOR CONTRIBUTIONS

Patricia Sansourekidou drafted the initial case text. Leonard Kim and Dongxu Wang provided critical revision and adaptation. Lee Xu, Mary Gronberg, and Cassandra Stambaugh provided critical inputs on LOs and the case text. All authors approved the final version.

## CONFLICT OF INTEREST

No conflict of interest.

## Supporting information

Supporting Information

## Notes

i Ellet W. The Case Study Handbook: A Student's Guide (Revised Edition); 2018.

ii The underscored terms are key words from MPLA Curriculum at https://w3.aapm.org/leadership/curriculum.php.
