# Supplementary material for: MPLA case: How do you lead as a lead physicist?
Source: J Appl Clin Med Phys. 2023 Apr 13;24(9):e13994. doi: 10.1002/acm2.13994 (PMC10476971; doi:10.1002/acm2.13994)
Supplement: Supplementary file 1 — Supporting Information [file ACM2-24-e13994-s001.pdf]

# Supplemental Materials

*Sample answers to the suggested discussion questions:*

1. David identified himself as a leader of not only the Red Sands medical physics team, but also a general leader to improve many other aspects of radiotherapy at Red Sands. As appointed by ULSCC, he also identified himself as first and foremost an employee of ULSCC responsible for change at Red Sands. This appears to align with ULSCC's expectation for him, that is, he'll bring Red Sands "in line with ULSCC". However, the role that is expected of David by ULSCC is not aligned with that of Red Sands. Red Sands is more likely to anticipate David becoming an essential part of their team before anything else. It's possible that by demonstrating an attitude of superiority towards the team at Red Sands, David hindered his ability to form genuine connections built on trust. These inconsistent and competing elements in David's role are the background of David's unsuccessful initial leadership work at Red Sands.
2. It is not uncommon for a person to have simultaneous but competing roles inside an organization. For example, David is expected by ULSCC to bring changes to Red Sands, but is likely expected by Red Sands to work as a clinical physicist, contributing to good care while bringing gradual improvement. It is critical for one to recognize the existence of competing roles. Once recognized, one could identify the priorities of these competing roles, and consider an optimal goal to accomplish. If an optimal win-win situation is not possible, then one should consider the trade-offs between these competing objectives. Last but not the least, in this specific case, David should also be aware of his formal employment relationship with ULSCC, explain his situation to his direct supervisor(s), and seek understanding and support for his plan.

3. Despite David's success with accreditation inspections at his previous institution, he should have first understood the existing system at Red Sands, which has also passed inspections and earned national accreditation year after year. Additionally, he used the inspection as an opportunity to change Red Sands' practice to be in line with ULSCC, which may not be the best combination of motivations. David also seemed to believe the official leadership role gave him the power to direct changes or as the chief therapist Mark put it, have his email be treated "as law". However, despite being a leader in title, he can only be truly effective at leading through influence and genuine persuasion. In his work of revising the policy and procedures, he didn't appear to have consulted and learned what was in the existing system, but directly provided his changes. He didn't communicate well in the actual rollout either; he relied on formal documentation, instead of communications, to introduce changes. At a minimum, staff training, in-service, and thorough explanation of the rationales should be provided to all affected staff. As a result, his unilateral actions were not viewed favorably and didn't receive buy-in from his colleagues at Red Sands.
4. David was not successful in influencing and persuading the chief therapist Mark to adopt new simulation procedures and immobilization devices. He attempted to do so by providing research papers and technical details, with the undertone of "I know better". To become more influential, David should first work on building rapport and earning trust with Mark (and others) and offer to help solve existing problems. For the issue of simulation without advanced immobilization devices, he should have assessed and better understood the clinical impact of the current and proposed methods. He should have also assessed the impact the changes would have on the therapists' workflow, patients' experience, etc., and considered mitigations if the proposed changes would

bring extra burdens to therapists. David could also have been more thorough in his approach, by either arranging a vendor presentation on the new immobilization devices or organizing a site visit to the ULSCC flagship hospital so the Red Sands physicians and therapists could observe the different procedures and devices firsthand. These approaches could reduce the perception of his personal desire in making those changes and be better perceived by the local team members.

5. There are many things David could have done differently. To name a few general areas: (1). He should develop better organizational awareness and become more sensitive to his role, perceived or entitled, at Red Sands and with ULSCC. A better organizational awareness can help him determine his priorities and maintain relationships with his supervisor at ULSCC and co-workers at Red Sands. (2). David should also start by learning the system at Red Sands, with a focus on serving his new clinic. He should refrain from judgment and eagerness for drastic changes until he becomes a trusted member of his new team. (3). He should consider the proper communication methods in his interactions. He should particularly avoid introducing changes as commands, because he assumes the role with the "Lead Physicist" title.
